# Phenotypic plasticity in growth and fecundity induced by strong population fluctuations affects reproductive traits of female fish

**DOI:** 10.1002/ece3.1936

**Published:** 2016-01-11

**Authors:** Juha Karjalainen, Olli Urpanen, Tapio Keskinen, Hannu Huuskonen, Jouko Sarvala, Pentti Valkeajärvi, Timo J. Marjomäki

**Affiliations:** ^1^ Department of Biological and Environmental ScienceUniversity of JyväskyläP.O. Box 35JyväskyläFI‐40014Finland; ^2^ MetsähallitusJyväskyläP.O. BOX 36JyväskyläFI‐40100Finland; ^3^ Natural Resources Institute FinlandSurvontie 9AJyväskyläFI‐40500Finland; ^4^ Department of BiologyUniversity of Eastern FinlandP.O. Box 111JoensuuFI‐80101Finland; ^5^ Department of BiologySection of EcologyUniversity of TurkuTurkuFI‐20014Finland

**Keywords:** Age of maturation, coregonids, embryonic development, fisheries, larval development, maternal effect, stock fluctuations

## Abstract

Fish are known for their high phenotypic plasticity in life‐history traits in relation to environmental variability, and this is particularly pronounced among salmonids in the Northern Hemisphere. Resource limitation leads to trade‐offs in phenotypic plasticity between life‐history traits related to the reproduction, growth, and survival of individual fish, which have consequences for the age and size distributions of populations, as well as their dynamics and productivity. We studied the effect of plasticity in growth and fecundity of vendace females on their reproductive traits using a series of long‐term incubation experiments. The wild parental fish originated from four separate populations with markedly different densities, and hence naturally induced differences in their growth and fecundity. The energy allocation to somatic tissues and eggs prior to spawning served as a proxy for total resource availability to individual females, and its effects on offspring survival and growth were analyzed. Vendace females allocated a rather constant proportion of available energy to eggs (per body mass) despite different growth patterns depending on the total resources in the different lakes; investment into eggs thus dictated the share remaining for growth. The energy allocation to eggs per mass was higher in young than in old spawners and the egg size and the relative fecundity differed between them: Young females produced more and smaller eggs and larvae than old spawners. In contrast to earlier observations of salmonids, a shortage of maternal food resources did not increase offspring size and survival. Vendace females in sparse populations with ample resources and high growth produced larger eggs and larvae. Vendace accommodate strong population fluctuations by their high plasticity in growth and fecundity, which affect their offspring size and consequently their recruitment and productivity, and account for their persistence and resilience in the face of high fishing mortality.

## Introduction

**Figure 1:**
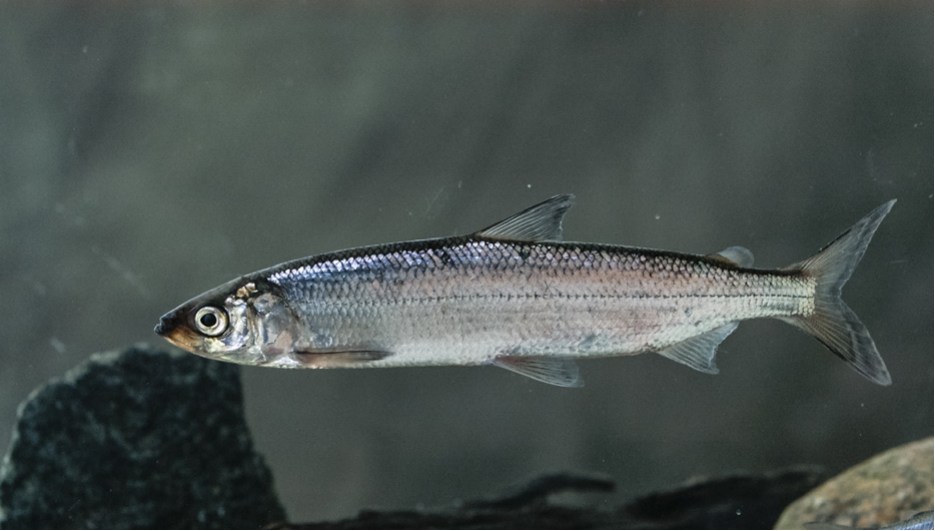
Our study species vendace (*Coregonus albula* (L.)). Photograph by Jussi Murtosaari.

Phenotypic plasticity occurs when the phenotype expressed by a given genotype changes as environmental conditions vary (Nussey et al. 2007). Fish are known for their high phenotypic plasticity in many life‐history traits in relation to environmental variability, and this is particularly pronounced among salmonids (Salmonidae) in the Northern Hemisphere (Skúlason and Smith 1995). Trade‐offs in phenotypic plasticity between life‐history traits related to the reproduction, growth, and survival of individual fish may have consequences for the age and size distributions of populations, as well as their dynamics and productivity (Stearns and Koella 1986; Heino et al. 2002). Age and size at maturation of fishes with moderate‐to‐high fecundity, high age at maturation, and low prematuration survival have been widely studied using the reaction norm approach (Heino et al. 2002; Evans et al. 2010; Jonsson et al. 2012; Kokkonen et al. 2015). Those species have high variation in age and size at maturation relative to certain salmonid species, such as vendace (*Coregonus albula* (L.), Fig. 1), with early maturation, high reproductive effort, small size, short life span, high demographic resilience, and low juvenile survivorship (Karjalainen and Viljanen 1994). Furthermore, growth of vendace is strongly dependent on the population density due to intraspecific food competition (Viljanen 1986; Helminen et al. 1992; Marjomäki and Kirjasniemi 1995; Auvinen et al. 2000), so that the wet mass of immature fish after the first growth season can vary by as much as fourfold between successive years (Helminen et al. 1992).

In vendace populations, intercohort competition has been proposed to induce a quantitative maternal effect on offspring quality during the growing season prior to spawning (Hamrin and Persson 1986). This interaction can be illustrated by the following chain of association: High young‐of‐the‐year abundance causes severe food competition between young‐of‐the‐year and mature fish which leads to slow growth and weakened condition of mature fish with deterioration in some essential quality or quantity components in the eggs. Reproductive products of poor quality will lead to a cohort effect of low survival of embryos or larvae, and thus less successful recruitment of the next year class. Further, a high mortality of vendace due to intensive harvesting will simplify the age structure of the spawning population from a multicohort iteroparous state to an almost totally single‐cohort semelparous state and maintains the induced 2‐year generation cyclicity in the population dynamics (Hamrin and Persson 1986) as well as the growth of individual fish (Helminen and Sarvala 1994). Thus, harvesting may significantly adjust the reproductive traits of individuals and populations in vendace, our model species.

Vendace is a special case among iteroparous fishes, because the age at the first reproduction of females in many vendace stocks has very little variation: Almost all females spawn for the first time in their second autumn (young, novice spawners at age of 1+) (Hamrin and Persson 1986; Huusko and Hyvärinen 2005), and only a small proportion of them survive to repeat spawning in the following years. However, some vendace populations with higher and more variable age at maturation are also known (Gregersen et al. 2011), and the spawning experience and age of a female may have a significant effect on the reproductive success of fish (Kamler et al. 1982; Evans et al. 1996; Busch et al. 2009).

We studied the effect of plasticity in growth and fecundity of vendace females on their reproductive traits using a series of long‐term incubation experiments. The wild parental fish originated from four separate populations with markedly different densities and hence lake‐specific naturally induced differences in their growth and fecundity. We interpreted here that the large differences in growth of parental fish were mainly caused by their phenotypic plasticity in response to the population density, and although local adaptations may evolve in fish populations rapidly (Eizaguirre et al. 2012; Westley et al. 2012), their role in our analysis is insignificant. The energy allocation to somatic tissues and eggs prior to spawning (variable *P*_S+R_) served as a proxy for total resource availability to an individual female, and its effects on offspring survival and growth were analyzed. Firstly, we examined whether the *P*_*S+R*_ differed between the females from the different lakes, the relative priority of growth and eggs in the energy allocation by young and old spawners, and how the *P*_*S+R*_ affected the egg production (relative fecundity, egg size) of females. Secondly, differences in survival and growth of offspring from young and old spawners with different *P*_*S+R*_ were studied using common garden fertilization and incubation experiments. Shortage in maternal food resources has been observed to increase the egg size via a trade‐off between egg number and size and subsequently to affect offspring size and survival (Hutchings 1991). Finally, we explored whether the allocation mechanisms revealed by our experiments can explain the high variability in the recruitment of vendace in our study lakes.

## Materials and Methods

### Study lakes and populations

Mature vendace to provide eggs and milt were caught from four Finnish lakes: Pyhäjärvi (I), Pyhäselkä (II), Puulavesi (III), and Southern Konnevesi (IV); hereafter the lakes are referred to by their roman numerals I–IV. The lakes were selected to represent the wide range of vendace population densities in Finland: The density of the mature adult (age ≥ 1+) vendace in 2004–2006 ranged from about 20 to 4000 individuals ha^−1^ in autumn, and the total biomass of vendace in autumn ranged from 12 to 42 kg·ha^−1^ (Table 1). The density estimates were based on the removal method (Helminen et al. 1993) and cohort analysis (Pope 1972) in the intensively fished Lake I and combined results of echo‐sounding and CPUE in Lakes III and IV (Marjomäki & Huolila 1995; Valkeajärvi and Marjomäki 2013). Data for adult fish from Lake II were scarce, and age‐specific density estimates were not available for the study years. However, from total catch statistics its density was roughly estimated to be the second lowest. Fishing mortality differed between lakes being highest in Lake I (Table 1, Appendix S1), which is shallower and more productive than the other lakes and has a longer open‐water period than the other lakes (Appendix S1).

**Table 1:** Characteristics of vendace populations [mean (SD)] in the study lakes in 2004–2007. Wet mass of females after first year in second spring was estimated from the scale by the Monastyrsky's equation (Monastyrsky 1930)

	Lake I	Lake II	Lake III	Lake IV
	SW Pyhäjärvi	Pyhäselkä	Puulavesi	S Konnevesi
Total catch, kg·ha^−1^	13 (1.0)	1 (0.2)	4 (0.5)	3 (0.5)
Total mortality, %	>90	–	50	50
Total density of spawners, ind. ha^−1^	157 (116)	–	1072 (353)	2770 (834)
Total biomass of spawners, kg·ha^−1^	10 (6)	–	15 (4)	18 (5)
Density 0+ in autumn, ind. ha^−1^	1063 (265)	–	953 (715)	1541 (1610)
Biomass 0+ in autumn, kg·ha^−1^	20 (9)	–	4 (3)	4 (4)
Total biomass in autumn, kg·ha^−1^	30 (10)	–	19 (5)	22 (9)
Wet mass of 1‐year old females, g	29 (8)	8 (3)	4 (1)	3 (2)
Wet mass of novice females, g	78 (22)	25 (6)	11 (2)	9 (5)
Wet mass of repeated spawners, g	127(48)	34 (10)	19 (5)	10 (3)
Total length of all mature females, mm	216 (24)	155 (18)	124 (14)	112 (23)
Fecundity of mature females, eggs	9791 (3966)	3593 (1317)	1739 (714)	999 (333)
% of mature individuals at age 1+	99 (1)	98 (2)	100	100
% of novice spawners	84 (9)	58 (36)	40 (45)	45 (32)
Density of hatched larvae, ind. ha^−1^	5865 (4590)	1520 (1021)	8726 (7038)	22105 (26036)

– = no data available.

The annual population fecundity was calculated by multiplying female (sex ratio assumed to be 1:1) spawning stock density (ind. ha^−1^) by the mean fecundity (eggs female^−1^) of mature females in each lake. The densities of newly hatched larvae of vendace (ind. ha^−1^) in spring 2004–2007 were estimated for all lakes using a stratified sampling design (Urpanen et al. 2009).

### Energy allocation by female vendace to somatic growth and eggs

Total energy allocated to somatic growth and eggs (*P*_*S+R*_) during the growing season (kJ·g^−1^ of fish wet mass) was estimated from randomly sampled ripe females (eggs not running). Total length (mm) and wet mass (g) of these females were measured. Both ovaries were removed and weighed to the nearest 0.01 g. Three subsamples taken from different parts of the gonads were weighed, and the number of eggs per subsample was counted under a dissecting microscope. Mean egg number of the subsamples was multiplied by the ratio of the total sample mass to the mean subsample mass to obtain an estimate of absolute fecundity of each female. Vendace is a determinate and total spawner species: The whole clutch of developed oocytes is laid down during a single period with no further recruitment of oocytes before the start of spawning (Dlugosz and Worniallo 1985; Murua et al. 2003). Thus, the oocyte number prior to spawning gives an adequate estimate of the total individual fecundity (Kjesbu et al. 2010) which makes it possible to estimate the total energy allocated to reproductive tissue. In our analysis, *P*_*S+R*_ was assumed to reflect the level of maternal food resources (indirectly) and growth conditions (directly), and the interannual variation in population density induced the variation in *P*_*S+R*._

Relative fecundity of females was calculated by dividing absolute number of eggs by total mass of fish including gonads (*W*_*T*_, wet mass). One subsample of unfertilized eggs from each female was frozen at −80°C for the determination of the energy content of eggs by their carbon content. Three pooled (five eggs) replicates from each female were weighed and freeze‐dried and then ground to a fine powder using a mortar and pestle. Approximately 0.6 mg of powdered sample was weighed into a small tin cup for the carbon analysis. Analyses were carried out using a FlashEA 1112 elemental analyzer connected to a Thermo Finnigan DELTAplus Advantage mass spectrometer. Carbon content was transformed to the energy content using a coefficient of 44.9 kJ·g^−1^ C (M. Paalavuo, P. Muje & J. Karjalainen, unpublished data).

Age of female vendace was determined from the scales and otoliths (sagittae), and the radius of each annulus in the scales was measured. Length at age *t*−1, the beginning of the last growing season, was back‐calculated using Monastyrsky's equation (Monastyrsky 1930), and the value of the exponent (*b *=* *0.607) was determined from our own data (*n *=* *817).

Total energy allocated to somatic growth and eggs (*P*_*S+R, i*_, kJ·g^−1^) was estimated individually for each female (*i*)

*P*_*S+R, i*_ = (*W*_*T, t, i*_ − *Ŵ*_*S, t*−1, *i*_) * *E*_*i*_/*Ẅ*_*t−*1*−t, i*_where

*W*_*T, t, i*_ = observed total wet mass at time *t* of female *i*,

*Ŵ*_*S, t*−1, *i*_ = estimated somatic wet mass at the beginning of the last growing season, at time *t*−1,

*E*_*i*_ = the gross energy content estimate (kJ·g^−1^) of female *i*.

*Ẅ*_*t−*1*−t, i*_ = mean weight of the female *i* during the last growing season.

Estimated somatic wet mass at time *t*−1 is

*Ŵ*_*S, t*−1, *i*_ = *a* * *Ĺ*_*t*−1, *i*_^*b*^ * [*W*_*T, t, i*_/(*c* * *L*_*t, i*_^*d*^)]where

*Ĺ*_*t*−1, *i*_ = back‐calculated length of female *i* at *t*−1,

*L*_*t, i*_ = its observed length at *t*,

*a* and *b* are the intercept and the slope of log‐linear regression of somatic wet mass (*W*_*S, t*_) on total length (*L*_*t,*_) of female vendace (*a *=* *0.0000044, *b *=* *3.06, *R* ^2^ = 0.98, *n *=* *461), respectively, and

*c* and *d* are the intercept and the slope of log‐linear regression of total wet mass (*W*_*T, t*_) on total length (*L*_*t*_) of female vendace (*c *=* *0.0000051, *d *=* *3.09, *R* ^2^ = 0.98, *n *=* *461), respectively.

The mean mass of the female *i* during the last growing season is

*Ẅ*_*t−*1*−t, i*_ = (*W*_*T, t, i*_ + *Ŵ*_*S, t*−1, *i*_)/2

*E*_*i*_ = the gross energy content (kJ·g^−1^) of female *i* is based on its *Ẅ*_*t−*1*−t, i*_.

*E*_*i*_ was estimated from log‐linear regression between gross energy content and *W*_*T*_ of age > 0+ vendace (*R* ^2^ = 0.937, *n *=* *861, M. Paalavuo, P. Muje & J. Karjalainen, unpublished data):

*E*_*i*_ = 4.231 * *Ẅ*_*t−*1*−t, i*_^0.112^

Energy allocation to eggs (*P*_*R*,* i*_, kJ·g^−1^) is estimated from

*P*_*R*,* i*_ = (*F*_*i*_ * *E*_*a*_)/*Ẅ*_*t−*1*−t, i*_where

*F*_*i*_ **= individual absolute fecundity and

*E*_*a*_ = mean energy content of egg at given age group *a*, and was determined for each age group from every lake and year from the carbon content of the eggs.

### Fertilization and incubation experiments

The experiments were carried out similarly in all periods 2004/2005, 2005/2006, and 2006/2007. All fish were caught by local fishermen in the middle of the spawning season from the last week of October to the first week of November. Fish were caught with trap nets (Lake I), gill nets (Lake II), and seine nets (Lakes III and IV) and before the fertilization fish were kept at the same temperature as the water temperature in the spawning area (2–3°C). Only live individuals from the catch with running milt and eggs were used for the fertilization experiment. Annually in each population, 9 ready‐to‐spawn females and 27 males were picked from a random sample of ca. 100 mature fish (Appendix S2). Both young (1+) and old spawners (> 1+) were included in the fertilizations. *L*_*T*_ and *W*_*T*_ of each fish were measured before removing the eggs and milt for the fertilization. Fertilization was performed immediately after fish were caught in the well‐stocked field laboratory established in a van. From each female, three sets of a minimum of 100 eggs were stripped into plastic Petri dishes. Each set was fertilized with a mixture of milt from three males to ensure sufficient milt and to simulate the communal spawning in nature. After adding the milt, a small volume of lake water was added to the dishes to activate the sperm and the dishes were gently waved to spread the sperm uniformly. The dishes were placed in one‐liter plastic bags with lake water at 2–5°C and transported within 2–4 h to a hatchery at the Konnevesi Research Station of the University of Jyväskylä, where the sets of eggs were distributed into incubation plates in randomized order and incubated in acrylic plastic plates (5 × 5 cm) with gentle continuous vertical water flow through their mesh bottom.

Fertilization success of eggs, that is, the proportion of fertilized eggs of all eggs (random sample of 50–100 eggs per set), was determined 24–48 h after fertilization under a dissecting microscope. Fertilized eggs were identified by visible cell division. Fungal growth was controlled by weekly treatment with malachite green until the embryos reached the eyed stage. Dead eggs were counted and removed weekly and water temperature recorded daily throughout the incubation period. Mean (± SD) water temperatures during incubations were 3.0 ± 1.1, 3.2 ± 1.0 and 2.7 ± 0.7°C during the incubation period in 2004/2005, 2005/2006, and 2006/2007, respectively. The total duration of the incubation period in different years, from fertilization to 90% hatching, was 170–196, 164–184, 171–194, and 171–195 days for eggs from Lakes I, II, III, and IV, respectively.

The instantaneous total mortality (*Z*) of embryos and larvae during experiments was calculated separately for each egg and rearing set:

*Z *=* *−ln (*N*_*e*_/*N*_*b*_),

where *N*_*e*_ = the number of live embryos or larvae at the end of a period and *N*_*b*_ = their number at the beginning of that period. A mean *Z* for each female was calculated as a mean of the three replicates (egg sets), and population‐specific estimates were calculated as a mean of the nine females. Mortality rates were calculated separately for two periods: *Z*_*F*_ = fertilization failure and *Z*_*E*_ = total embryonic mortality from fertilization to 100% hatching (including *Z*_*F*_ and *Z*_*L*_). Values of proportional total mortality (*A*, %) were calculated from

*A *=* *1 – exp(‐*Z*).

The proportion of hatched larvae alive (*H*%) was also recorded.

The mean egg wet mass for each of the nine females per lake was measured from 50 eggs (Appendix S2). During the hatching period, the number of dead eggs and hatched larvae was recorded daily. One subset (*n *=* *10) of hatched larvae from each female was preserved in 70% ethanol for the measurements of *L*_*T*_ and *W*_*T*_.

### Larval growth, survival, and swimming performance experiments

Hatched larvae from the laboratory incubations were moved daily to flow‐through tanks (26 liters) and larvae from each lake and from 3 to 5 females were kept separately in the collecting tanks for a maximum of 3 days without food in order to collect enough larvae for rearing experiments. Subsequently, newly hatched larvae were divided into aerated flow‐through aquaria (length 40 cm × width 25 cm × height 20 cm), and 65 larvae per aquarium with 2–5 replicates per lake were reared for 23 days to monitor their growth and mortality. The photoperiod during rearing experiments was set at 18‐h light: 6‐h dark, and the larvae were fed *ad libitum* on hatched *Artemia* nauplii without cysts (daily ration adjusted to 100% of the *W*_*T*_ of the larvae). The aquaria were cleaned daily by siphoning out feces and uneaten *Artemia*. The number of dead larvae and water temperature were recorded daily. The instantaneous mortality (*Z*_*L*_) during the larval rearing period was calculated separately for each aquarium, and the lake‐specific *Z*_*L*_ was calculated as a mean of aquaria for a given population. The mean (± SD) rearing temperatures were 12.9 ± 0.4, 13.5 ± 0.6 and 15.0 ± 0.4°C in spring 2005, 2006, and 2007, respectively.

A random sample of five larvae from each aquarium was taken for a challenge test. Challenge tests were performed in a flow‐through swimming chamber with a volume of 18 ml. The chamber was connected by rubber hoses to a water circulation pump, and the whole system was immersed in an aquarium. Water velocity in the chamber was calibrated separately in each trial according to the average *L*_*T*_ of larvae at a given time. Five larvae were placed in the swimming chamber, and water flow was adjusted to the level of 1 *L*_*T*_·s^−1^ for 1 min, after which water velocity was increased to 5 *L*_*T*_·s^−1^ (SD = 1). Swimming ability of larvae against flow was recorded as the time (s) until the median larva (third of the five) was exhausted and was unable to keep its position in the swimming tunnel. After that, larvae were removed from the chamber and anaesthetized before measurement of individual *L*_*T*_ and *W*_*T*_.

### Data analysis

A general linear model (*GLM*) with a main effect model structure was used to test for the effect of lake, study year, and age of spawners (young vs. old spawners) on the total energy allocated to growth and eggs (*P*_*S+R*_, kJ·g^−1^) or to reproduction (*P*_*R*_, kJ·g^−1^) of females in the summer before spawning. Wet body mass of the females was used as a covariate in the models.

*GLM,* with *P*_*S+R*_ as a covariate, was used to test for the effect of the spawning history on the relative fecundity (eggs g^−1^) and wet mass of eggs (mg) of females. In these analyses, the *P*_*S+R*_ of individual fish from the study lakes was assumed to reflect the lake‐ and year‐specific status including the thermal effects, so lake and year factors were no longer included in the models.

*GLM,* with the total biomass of vendace (kg·ha^−1^) in summer before the spawning season as a covariate, was used to test for the effect of the female age (young vs. old) on *P*_*S+R*._

The difference in the fertilization failure (*Z*_*F*_), total mortality of embryos during the experimental incubation period (*Z*_*E*_), proportion of hatched larvae alive (*H*%), and *L*_*T*_ and *W*_*T*_ of the hatched larvae were compared between young and old spawners by the *GLM*s with *P*_*S+R*_ as a covariate.

In larval experiments, the rearing temperature differed between the study years due to problems with the heating system. Thus, the temperature effect on the growth of the larvae was corrected by a temperature‐specific growth function derived from the experimental data of Luczynski (1991). The *L*_*T*_ and *W*_*T*_ of larvae in each aquarium were adjusted to correspond to growth at 13.5°C which was the mean rearing temperature in 2006. Because the larvae of all females from a lake were pooled in the rearing experiments (not possible to identify mothers of individual larvae), the *P*_*S+R*_ for the analysis was calculated as an annual mean for all females in specific year and lake. Data were divided further into two *P*_*S+R*_ classes lower and higher than the overall mean for the females in all years (overall mean = 4.6 kJ·g^−1^). The differences in temperature‐corrected *L*_*T*_ and *W*_*T*_ were tested by *t*‐test between the two *P*_*S+R*_ classes. *GLM* model with the ln‐transformed rearing temperature as a covariate was used to test the effects of *P*_*S+R*_ on the *Z*_*L*_ and swimming performance of larvae after the 23‐days rearing experiment. In this analysis, *P*_*S+R*_ class was a fixed factor.

## Results

### Plasticity in growth and fecundity

**Figure 2:**
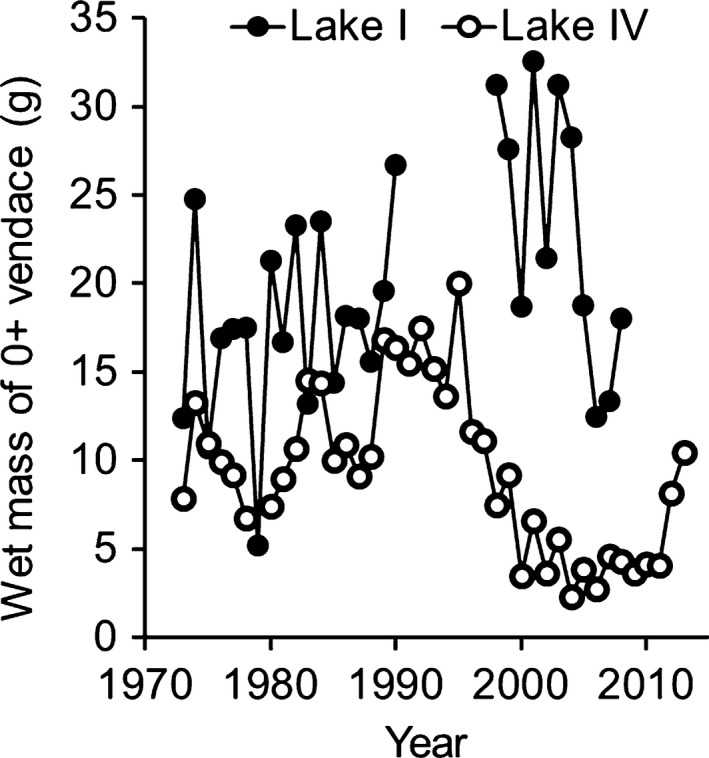
Interannual variation in the mean wet mass (g) of 0+ vendace in 1973–2013 (Helminen et al. 1992) in Lake I and Lake IV (P. Valkeajärvi, unpublished data).

The large differences in the population density between lakes caused high variation in the mean size of both juvenile and mature fish, as well as in the fecundity of females. The interannual variation in the wet mass of 0+ fish after the first growing season was high, and even a fourfold difference between successive years has been observed in Lake I (Fig. 2). The majority of vendace females spawned for the first time in their second autumn (Table 1, % of mature individuals at age of 1+) and the proportion young spawners varied between 40 and 84% of all spawners. Thus, every year there were also older and likely repeat spawners (age 2+ or older) present in all lakes, but their proportion and total number were clearly lowest in Lake I with the lowest population density and highest adult mortality due to intensive fishing.

**Figure 3:**
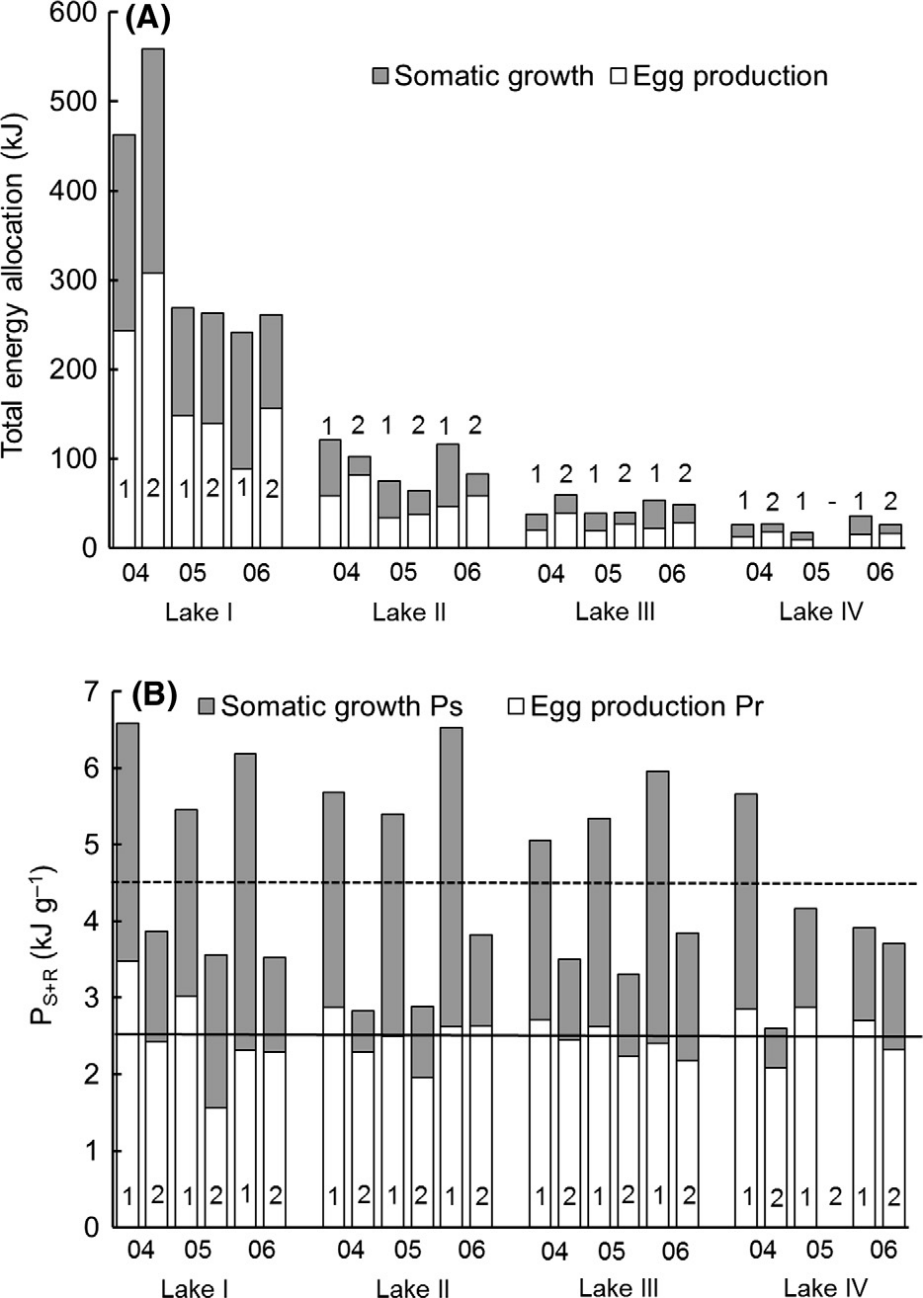
(A) Total energy allocation to somatic growth (kJ) and eggs (kJ) by young (1) and old (2) spawners in Lakes I–IV. (B) Relative energy allocation to somatic growth (*P*_*S*__,_ kJ·g^−1^ of female wet mass) and egg production (*P*_*R*_, kJ·g^−1^ of female wet mass) by young and old spawners in Lakes I‐IV. The means over all lakes and years for total energy (*P*_*S*__*+R*_, kJ·g^−1^ of female wet mass) used for growth and egg production during growing season and for mean energy used for eggs (*P*_*R*_) are shown by dashed and solid lines, respectively.

Total energy (kJ) allocated by vendace females to somatic growth and eggs differed considerably between lakes (Fig. 3A) demonstrating the extreme variability of their growth and fecundity. Young spawners (age group 1+) invested significantly more (*F *=* *659.6, *P *<* *0.001, Fig. 3B) energy into growth and eggs (*P*_*S+R*_, kJ·g^−1^) than old spawners (age group ≥ 2+). The *P*_*S+R*_ also differed significantly between lakes (*F *=* *7.2, *P *<* *0.001), being largest in the sparsest population with extremely fast‐growing individuals in Lake I, and also between years (*F *=* *28.1, *P *<* *0.001). The effect of the covariate wet mass was not significant (*F *=* *0.01, *P *>* *0.05).

The energy allocated to eggs (*P*_*R*_, kJ·g^−1^) also differed significantly between young and old spawners (*F *=* *25.3, *P *<* *0.001). The *P*_*R*_ of young spawners was higher than that of old spawners (Fig. 3B), but old spawners allocated a higher proportion (69%) of total energy for eggs than young females (49%), and thus, a smaller share remained for somatic growth. Altogether, the variation in *P*_*R*_ was very low compared to that of *P*_*S*_. The *P*_*R*_ differed significantly between years (*F *=* *3.2, *P *<* *0.05) but did not differ between lakes (*F *=* *1.0, *P *>* *0.05). Again, the effect of the covariate mass was not significant (*F *=* *1.1, *P *>* *0.05).

**Figure 4:**
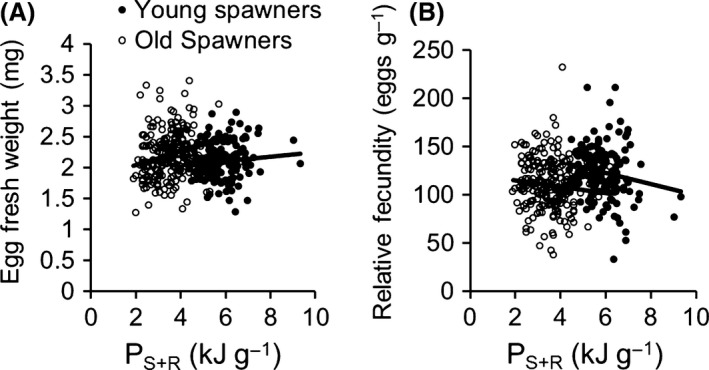
(A) Relationship between egg wet mass (g) and total energy allocated to growth and egg production (*P*_*S*__*+R*_, kJ·g^−1^ of female wet mass) during the growing season. Regression lines (*y* = *a* + *bx*) for young (*a* = 1.81 ± 0.12 (± SE), *b* = 0.039 ± 0.021) and old spawners (*a* = 1.83 ± 0.13, *b* = 0.11 ± 0.04) are given separately. (B) Relationship between relative fecundity of females (egg g^−1^ female wet mass) and *P*_*S*__*+R*_. Regression lines for young (*a* = 130.3 ± 11.1 (± SE), *b* = −2.53 ± 1.93) and old spawners (*a* = 121.7 ± 8.52, *b* = −3.31 ± 2.39) are given separately.

The large differences in the age‐specific size of females between lakes yielded large between‐lakes differences in their absolute fecundity (eggs female^−1^, Table 1). At the same size, the old spawners had on average 10% lower absolute fecundity than the young spawners. Wet mass of eggs (mg) and relative fecundity (eggs g^−1^ female wet mass) differed significantly between these spawning history classes (*F *=* *3.9, *P *<* *0.001; and *F *=* *25.9, *P *<* *0.001, Fig. 4). The egg mass of old spawners was higher but their relative fecundity was lower than those of young spawners. In the *GLM* model, the covariate *P*_*S+R*_ significantly affected the egg wet mass (*F *=* *11.5, *P *<* *0.01) and relative fecundity (*F *=* *3.6, *P *=* *0.06) of females: In both young and old spawners, higher *P*_*S+R*_ (and thus more energy available) was associated with higher egg mass but lower relative fecundity.

### Effects of *P*_*s+r*_ on fertilization failure and egg mortality

*P*_*S+R*_ had no effect on the fertilization failure *Z*_*F*_ (*F *=* *0.15, *P *>* *0.05) or the total embryonic mortality from fertilization to 100% hatching *Z*_*T*_ (*F *=* *0.15, *P *>* *0.05). Neither *Z*_*F*_ (*F *=* *0.15, *P *>* *0.05) nor *Z*_*T*_ (*F *=* *0.7, *P *>* *0.05) differed significantly between young and old spawners. In the incubation experiments, *Z*_*T*_ was generally high and variable between years and populations; the relative total mortalities (A%, mean ± SD) were 82 ± 9%, 53 ± 23%, 25 ± 16%, and 76 ± 36% in Lakes I, II, III, and IV, respectively. Fertilization failure constituted most of the total mortality.

### Effects of *P*_*s+r*_ on larval growth, mortality, and swimming performance

*P*_*S+R*_ had a significant positive effect on the total length (*L*_*T*_) of hatched larvae (*F *=* *4.3, *P *<* *0.05) but not on the proportion of hatched live larvae alive *(H*_*%*,_ *F *=* *2.7, *P *>* *0.05). *L*_*T*_ of newly hatched larvae (*F *=* *5.6, *P *<* *0.05) and *H*_%_ (*F *=* *7.5, *P *<* *0.05) differed significantly between young and old spawners. The mean *L*_*T*_ was 8.5 mm (SD = 0.7, *n *=* *31) and 8.7 mm (SD = 0.4, *n *=* *48) for the young and old females, respectively. The mean *H*_%_ was 93% (SD = 15, *n = *38) and 98% (SD = 7, *n *=* *59) for the young and old females, respectively.

**Figure 5:**
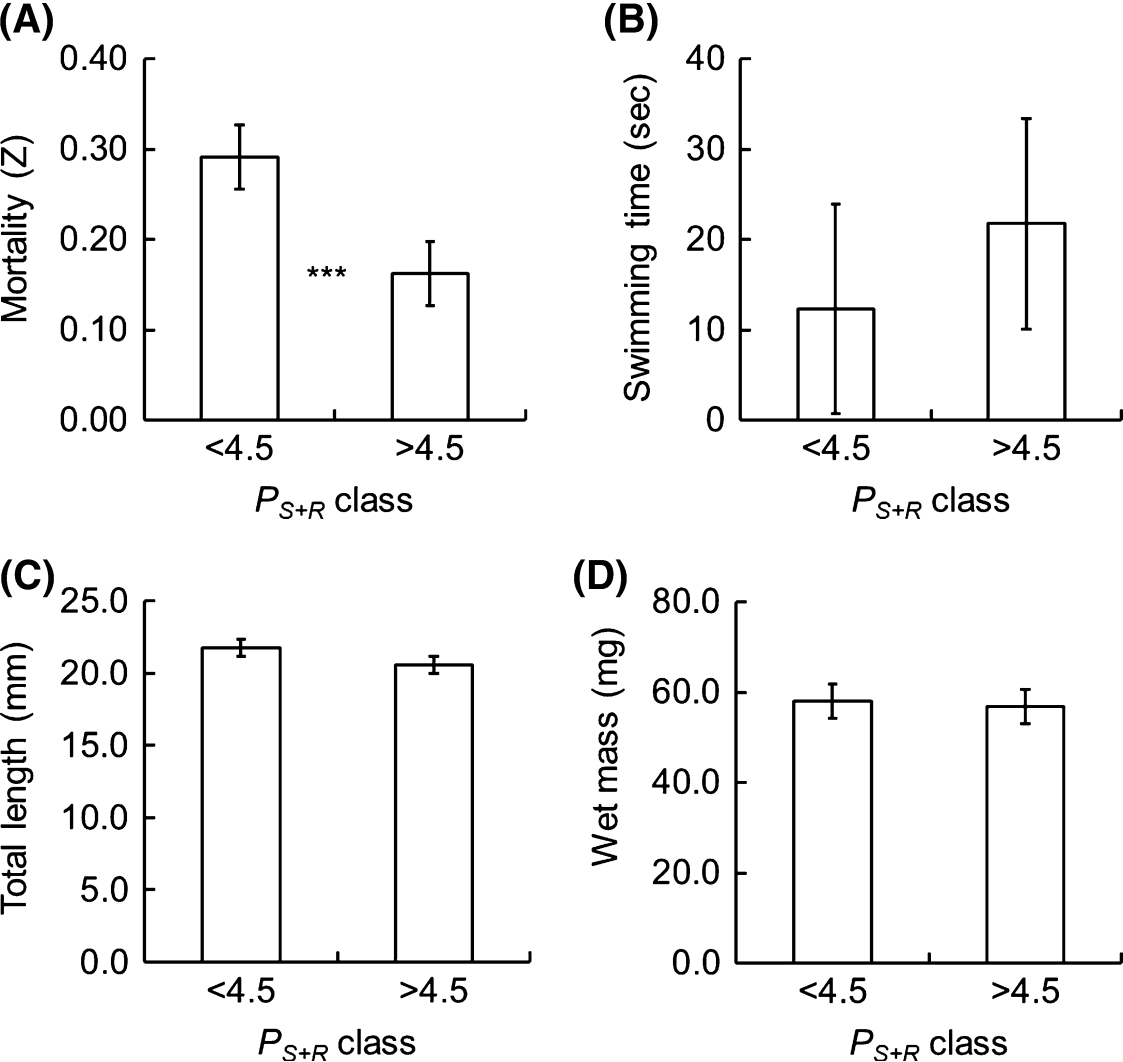
(A) Mortality (*Z*_*L*_), (B) swimming performance (seconds), (C) total length (*L*_*T*_, mm), and (D) wet mass (*W*_*T*_, mg) of vendace larvae at the end of the 23‐day rearing experiments in spring 2005, 2006, and 2007. Bars indicate the mean values of larvae hatched from the eggs of females with low or high *P*_*S*__*+R*_. Error bars represent the standard error of the mean, and ***indicates statistically significant difference between the low and high *P*_*S*__*+R*_.

After the 23‐days rearing experiment, the larval period mortality *Z*_*L*_ (Fig. 5A) differed between the low and high *P*_*S+R*_ classes (*F *=* *20.3, *P *<* *0.001) and the temperature covariate was also significant (*F *=* *13.8, *P *<* *0.01). Mortality in the low *P*_*S+R*_ class was higher than in the high *P*_*S+R*_ class. The swimming performance (Fig. 5B) of the larvae did not differ significantly between *P*_*S+R*_ classes (*F *=* *1.3, *P *>* *0.05), and the effect of temperature covariate was insignificant (*F *=* *0.5, *P *>* *0.05). There was no significant difference between *P*_*S+R*_ classes (Fig. 5C, D) in larval *L*_*T*_ or *W*_*T*_ (*t *=* *1.75, *P *>* *0.05; and *t *=* *0.24, *P *>* *0.05, respectively).

### Implications for population level

**Figure 6:**
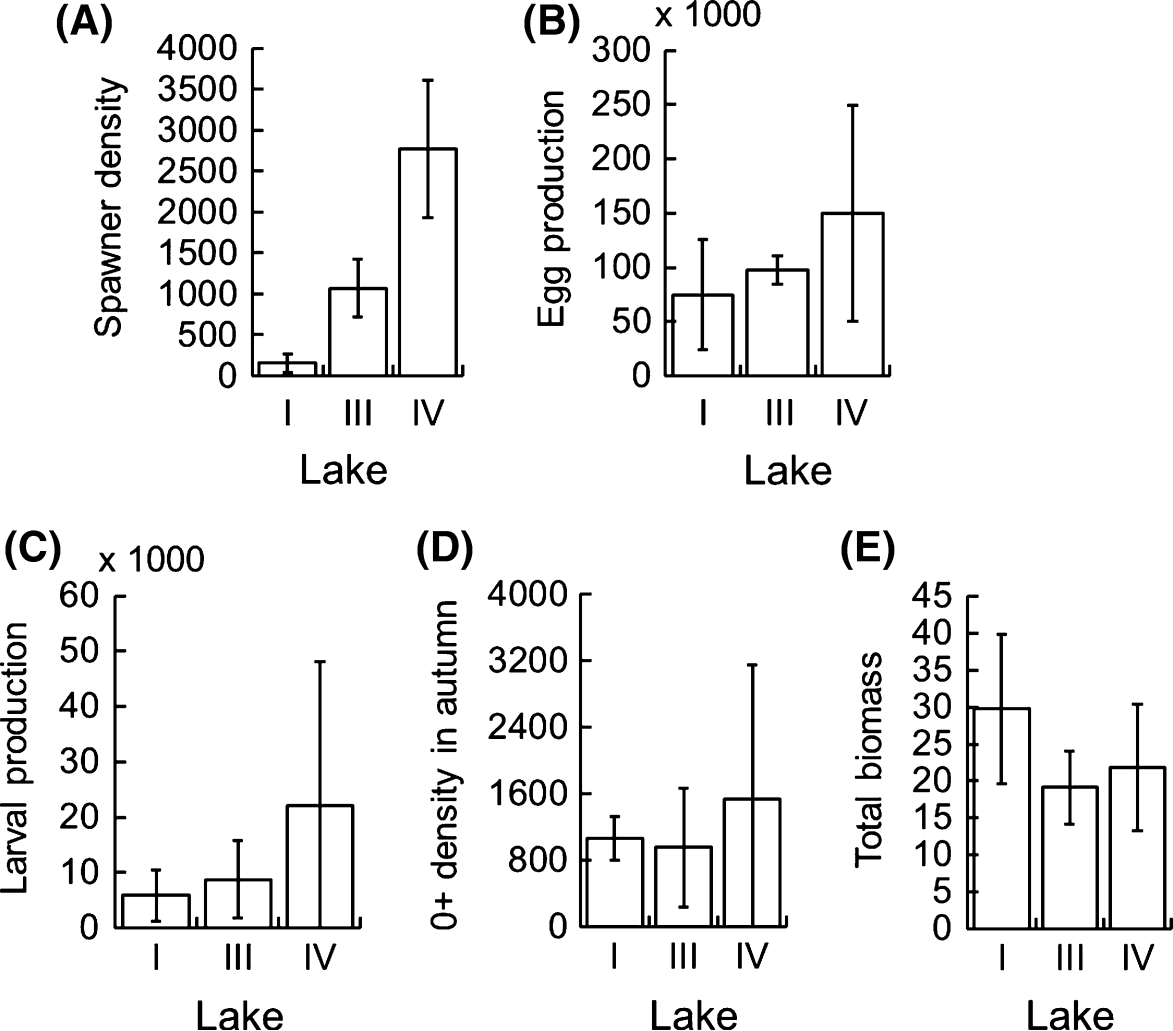
Density of spawning stock (A, mature individuals ha^−1^), population fecundity (B, eggs ha^−1^), larval density (C, larvae ha^−1^), density of 0+ vendace in autumn (D, individuals ha^−1^), and total biomass of vendace in autumn (E, biomass of 0+ and mature fish kg·ha^−1^) in the study lakes in 2003–2007. Bars represent annual means and vertical lines standard deviations.

In the study years, the estimates of mean density of spawners, egg production, and larval production differed considerably between the lakes (Fig. 6A–C): The offspring production was highest in Lake IV with the highest total density estimate, while the lowest egg and larval production was observed in Lake I with the lowest population density and the highest annual mortality. Instead, the mean annual density of 0+ fish in autumn showed low variation between the lakes (Fig. 6D), and the total annual biomass of the population showed little variation between the lakes or even an opposite trend compared to the density, with Lake I having the highest mean biomass among the study lakes (Fig. 6E).

## Discussion

In our study lakes, vendace females allocated a rather constant and high proportion of their energy investments to eggs (kJ·g^−1^) despite very different growth patterns depending on the resources available in the different lakes (e.g., range of mean *W*_*T*_ and fecundity at age of first reproduction from 5 to 132 g and from 530 to 21,000 eggs female^−1^, respectively). The investments into eggs thus dictated the share remaining for growth. Egg production by larger females and old spawners took a larger proportion of their total energy allocated to growth and eggs (*P*_*S+R*_) than the egg investments by smaller and young spawners, which led to a trend of decreasing growth rate at higher size and age. Such reduced growth after maturation is a phenomenon widely observed among fishes (Roff 1983; Folkvord et al. 2014), and although some fish species with limited food supply may partly or fully sacrifice egg production to preserve body condition, most maintain investment in reproduction at the expense of body reserves (Saborido‐Rey and Kjesbu 2005).

The energy allocation to eggs per body mass was higher in young than in old spawners, and the egg size and consequently the relative fecundity also differed, with young females producing more but smaller eggs so that the newly hatched larvae of young females were smaller than the larvae of old spawners. The recognized high value of large mature fish (high fecundity, large eggs, higher survival of offspring) to the populations of several species has led to the recommendation in their fisheries management to conserve large, typically old individuals (Duarte and Alcaraz 1989; Berkeley et al. 2004; Saborido‐Rey and Kjesbu 2005; Enberg et al. 2011). However, in our vendace experiments, the fertilization rate and the survival of embryos were not affected by the age of spawners or the total energy allocation to the eggs. This contrasts with the results of earlier experiments by Kamler et al. (1982) who reported that vendace females spawning for the first time produced the “worst” eggs, middle‐aged females produced the best eggs, and old spawners laid eggs with again lower quality. The embryonic mortality in our experiments was highest during early embryogenesis until the eyed stage, which has also been reported in previous studies (Wilkonska and Zuromska 1982; Kamler 2005; Karjalainen et al. 2014). Our fertilization procedure simulated the communal spawning whereby several males release their sperm simultaneously during spawning, and therefore, the paternal effects on embryonic survival remain unresolved.

In many fish species, the important reproductive traits of age and size at maturation vary with changes in population density (Adams 1980; Trippel 1995; Saborido‐Rey and Kjesbu 2005; Roos et al. 2006). As the age at maturation of vendace in Finnish lakes is practically fixed to their second autumn (age group 1+ years), the size at maturation varies markedly with the individual growth rate of fish, which again depends on population density. In our study lakes, fluctuation between high and low stock densities are typical and caused by interannual variation in environmental conditions (Karjalainen et al. 2000; Marjomäki 2003) as well as interstage effects of vendace populations (Marjomäki et al. 2014). In some Finnish lakes, intensive fishing of the vendace population appreciably shapes the spawning stock structure (Sarvala and Helminen 1995; Huusko and Hyvärinen 2005) and reproductive traits of the species. Especially in Lake I, a sparse population following high fishing mortality responds to high resource availability by high growth rate prior to spawning and subsequent high individual fecundity. A similar response of growth (Fig. 2) and fecundity has been seen in many other lakes, including Lake IV where in the 1990s a long and probably environmental‐induced phase of low vendace stock prevailed (Valkeajärvi and Marjomäki 2013).

Interestingly, the relative fecundity of vendace females in the sparse population was lower and egg size larger than those for fish growing in the dense stock with very much lower food ratio per capita. This response of egg size to food scarcity was opposite to the mechanisms reported among periodic salmonids, which tend to produce larger eggs if females undergo food scarcity prior to spawning (Thorpe et al. 1984; Hutchings 1991; Burton et al. 2013). This trade‐off of investing in higher quality, larger larvae while reducing their quantity under food limitation has been considered a beneficial adaptation in low resource environmental conditions (Hutchings 1991; Gregersen et al. 2011; Vrtilek and Reichard 2014). However, studies have revealed that the expression of maternal effects is context‐dependent and restricted maternal food availability has been shown to either decrease (Gagliano and McCormick 2007) or increase (Hutchings 1991; Guisande et al. 1996; Allen et al. 2008) offspring size and survival. Plaistow and Benton (2009) concluded that maternal effects in high competition population (low food ration) impact more on juvenile survival compared with low competition populations (high food ration), where maternal effects have more impact on the population growth rate.

In addition to the compensatory density‐dependent increase in prerecruitment survival of vendace along with the decrease in spawning stock (Marjomäki 2003), exceptionally high compensatory plasticity in growth and subsequent changes in fecundity and egg size increase the capacity of vendace populations to withstand high fishing mortality. In a sparse stock, females produce per capita more and larger eggs and larvae with higher survival: In Lake I, the spawning stock density was much lower than in the other lakes, but the estimate of survival from newly hatched larvae to 0+ fish in autumn was as high as 32 ± 30% for lake I (mean ± SD) compared to 11 ± 7% for Lakes III and IV. Moreover, the extremely high growth rate of fish in the Lake I prior to spawning resulted in total biomass of vendace in autumn as high as in the other lakes (Fig. 6E). The longer growing season and higher productivity in Lake I than in the other lakes also contribute to the high growth and egg production of vendace females, although the meta‐analysis by Viljanen (1986) showed that differences in the growth rate of vendace populations between southern and northern lakes was much smaller than the interannual differences in growth of fish in same lake and even in successive years.

Size‐selective fishing is commonly interpreted as one of the major causes behind observed changes in age at maturation by affecting growth rates and inducing evolutionary regime shifts (Saborido‐Rey and Kjesbu 2005; Roos et al. 2006). In vendace populations, fishing is reflected in other reproductive traits, and intensive harvesting has also been observed to simplify the spawning population structure causing changes in the population dynamics (Huusko and Hyvärinen 2005).

Although the large interannual differences in growth of parental fish within a lake were caused by their extreme phenotypic plasticity in response to the changes in the population density, we cannot totally overrule the effect of local adaptation, which has been suggested to be common in salmonids (Taylor 1991; Primmer 2011). Local adaptation of fish can be rapid, progressive process driven by environment‐induced selection process (Eizaguirre et al. 2012; Westley et al. 2012). Plasticity itself “is heritable and population differences in reaction norms can reflect adaptive responses, by natural selection, to local environments” (Hutchings 2011), and thus, locally adapted populations may vary in their plasticity. Our study species has high interannual variation in growth having large differences in mass of 0+ fish even between successive years, and the potential for plasticity seems to be similar in the study lakes (see Fig. 2). Also stock transfers of vendace by fisheries managers from lakes with dense population and slow‐growing individuals to another lake with sparse population has caused remarkable changes in growth rate of transferred fish matching with the amplitude of variability in our study lakes (Huuskonen, H., Marjomäki, T. J., unpublished results).

In the incubation experiments, the embryos of all populations developed at similar rates and their hatching success was high. The newly hatched larvae of old spawners were slightly larger than those of young spawners. According to the concept of “rapid growth leads to reduced susceptibility to size‐selective mortality and enables high survival and high fitness” (Miller et al. 1988) even small differences in the hatching size may significantly affect the future survival. Although Urpanen et al. (2005) observed no clear size‐dependent mortality during the early life of vendace in Finnish lakes, Auvinen (1995) and Sarvala and Helminen (1995) have reported that the year‐class strength of vendace in their study lakes seemed to be positively associated with egg size. In our rearing experiments, the mean lake‐specific instantaneous mortality rate (*Z*_*L*_) ranged from 0.10 to 0.34 and mortality did not differ among populations but was significantly different between the offspring of females with low and high *P*_*S+R*_. However, the growth and swimming performance of the larvae under experimental conditions did not differ between *P*_*S+R*_ classes. Although our experimental and field data basically support the asymmetric food competition hypothesis of Hamrin and Persson (1986) as a mechanism explaining the 2‐year cyclicity of vendace populations, the differences in larval survival and properties are far too small to explain substantially the dramatic 2‐year population fluctuation observed in some vendace populations.

As also observed among other fishes (Burton et al. 2013; Jonsson and Jonsson 2014), the growth history of vendace already during the first growing season and then during the maturation year seems to affect their fecundity and egg size. However, it is still unclear when the number of the mature eggs in the ovaries is determined. Oocyte growth and development in teleost fishes is an interactive process that is able to adjust to the prevailing conditions (Tyler and Sumpter 1996; Lubzens et al. 2010). In salmonids, oocyte development from the primary oocyte growth phase to the maturation and ovulation takes several months. Ovaries of vendace start to develop already in early spring (March–April) and vitellogenesis begins in July continuing until ovulation and spawning in November–December (Dlugosz and Worniallo 1985). During vitellogenesis eggs will be loaded with lipids and other reserves needed for embryogenesis after fertilization (Wiegand 1996; Lubzens et al. 2010), and the size of eggs will increase up to ovulation (Dlugosz and Worniallo 1985). Primordial germ cells and first‐stage oogonia can be observed in coregonids already at the juvenile stage (Krol et al. 2003), and probably the growth rate of fish in their first growing season gives the size‐dependent baseline for the fecundity (Lubzens et al. 2010; Gregersen et al. 2011). However, the number of oogonia is not finite, and dividing oogonia persist in the adult ovary to mid vitellogenesis (Tyler and Sumpter 1996). Hence, we must assume that both the final number and size of eggs can still be adjusted during the growing season prior to spawning, thus ensuring that the relative energy allocation to offspring is rather constant from year to year despite the vast and unpredictable variability in population density and thus in individual growth during the growing season prior to spawning.

Altogether, the density‐dependent growth of vendace influences egg production and recruitment of offspring and regulates the variability observed in many vendace populations. These small‐sized schooling fish compensate strong population fluctuations by their extreme plasticity in growth and fecundity, which affect their offspring size and consequently their productivity, and account for their persistence and resilience in the face of high fishing mortality. The variability in body size of short‐lived pelagic schooling fish under different environmental and nutritional conditions is exceptional among vertebrates and a special feature is also their capability to keep the relative fecundity constant regardless of high interannual fluctuation in their food resources per capita. Thus, the basis for the ecologically sustainable use for the populations of these commercially important species with dense schools is in many ways different compared to several other exploited species with later maturation, long life cycle, and different social structure of the population.

## Conflict of Interests

None declared.

## Supporting information


**Appendix S1**. Characteristics of study lakes in 2004–2007 (from databases of Finnish Environment Institute).

**Appendix S2**. Total number of fish, eggs and hatched larvae in experiments and measurements in different incubation periods in 2004–2007.
