# Supplementary material for: Phenotypic plasticity in growth and fecundity induced by strong population fluctuations affects reproductive traits of female fish
Source: Ecol Evol. 2016 Jan 11;6(3):779–90. doi: 10.1002/ece3.1936 (PMC4739574; doi:10.1002/ece3.1936)
Supplement: Supplementary file 1 — Appendix S1. Characteristics of study lakes in 2004–2007 (from databases of Finnish Environment Institute). [file ECE3-6-779-s001.docx]

Supporting information Appendix S1. Characteristics of study lakes in 2004-2007 (from databases of Finnish Environment Institute). Epilimnetic temperature (°C) is the mean temperature during open-water season (from May to October) in the depth of 0-10 m.

|  | Lake I | Lake II | Lake III | Lake IV |
| --- | --- | --- | --- | --- |
|  | SW Pyhäjärvi | Pyhäselkä | Puulavesi | S Konnevesi |
| Location | 60°54´ - 61°06´N 22°09´ - 22°25´E | 62°22´ - 62°38´N 29°32´ - 29°55´E | 61°41´ - 61°58´N 26°26´ - 26°52´E | 62°30´ - 62°40´N 26°20´ - 26°44´E |
|  |  |  |  |  |
| Area, km^2^ | 155 | 263 | 190 | 120 |
| Mean depth, m | 5 | 9 | 12 | 13 |
| Maximum depth, m | 26 | 67 | 69 | 56 |
| Total phosphorus, mg m^-3^ | 17 | 12 | 4 | 8 |
| Colour, Pt mg l^-1^ | 15 | 64 | 18 | 25 |
| Secchi depth, m | 3 | 2 | 5 | 5 |
| Chlorophyll *a*, mg m^-3^ | 6 | 7 | 3 | 3 |
| Epilimnetic temperature, ºC | 14.4 | 13.6 | - | 13.1 |
| Ice-free period, days | 223 | 208 | 215 | 208 |

Description of vendace fisheries in study lakes

The Lake I has supported very efficient vendace fishery. Annual winter seine catches have ranged from 2 to 24 kg ha^-1^ and typically majority of 0+ vendace have been removed during the first winter fishing season after recruitment (Helminen *et al*. 1993; Ventelä *et al*. 2007). In 2004–2007, the vendace population in the Lake I was sparse and the mean wet mass of 1-yr old females was almost ten-fold compared to the the mean of 3 g in the Lake IV (Table 2). Helminen, Sarvala & Karjalainen (1997) observed two-year cyclicity in the abundance of 0+ fish in 26-year data of this population. In the northernmost Lake II, the population of vendace has also been sparse and the mean wet mass of 1-year old females was 8 g in 2004–2007. In the Lake III, vendace population has fluctuated considerably (Marjomäki & Huolila 1995, 2001; Marjomäki *et al*. 2014) being rather dense during the study years. The growth of vendace has been strongly density-dependent (Marjomäki & Kirjasniemi 1995) and a tendency for two-year cyclicity has also been detected (Marjomäki *et al*. 2014). In the Lake IV, the vendace population has fluctuated strongly since 1970’s with a long low population period from the mid 1980`s to the mid 1990’s (Valkeajärvi & Marjomäki 2004, 2013). In 2003–2007, the vendace population was very dense, fish were small and the two-year cycle in year class strength was strong already at hatching.

Helminen, H., Ennola, K., Hirvonen, A. & Sarvala, J. (1993) Fish stock assessment in lakes based on mass removal. *Journal of Fish Biology*, 42, 255–263.

Helminen, H., Sarvala, J. & Karjalainen, J. (1997) Patterns in vendace recruitment in Lake Pyhäjärvi, south-west Finland. *Journal of Fish Biology*, 51, (Supplement A), 303–316.

Marjomäki, T.J. & Huolila, M. (1995) Monitoring the density of Lake Puulavesi vendace (*Coregonus albula* (L.)) by hydroacoustics, catch per unit effort, virtual population and catch per swept area. *Archiv für Hydrobiologie Special Issues of Advances in Limnology* 46, 267–276.

Marjomäki, T.J. & Huolila, M. (2001) Long-term dynamics of pelagic fish density and vendace (*Coregonus* *albula* (L.)) stock in four zones of a lake differing in trawling intensity. *Ecology of Freshwater Fish*, 10, 65–74.

Marjomäki, T.J. & Kirjasniemi, J. (1995) Density dependent growth of vendace (*Coregonus albula* (L.)) in Lake Puulavesi: a modelling analysis. *Archiv für Hydrobiologie Special Issues of Advances in Limnology*, 46, 89–96.

Marjomäki, T.J., Urpanen, O. & Karjalainen, J. (2014) Two-year cyclicity in recruitment of a fish population is driven by an inter-stage effect. *Population Ecology* 56, 513–526. doi 10.1007/s10144-014-0439-0.

Valkeajärvi, P. & Marjomäki, T.J. (2004) Perch (*Perca fluviatilis*) as a factor in recruitment variations of vendace (*Coregonus albula*) in Lake Konnevesi, Finland. *Annales Zoologici Fennici*, 41, 329–338.

Valkeajärvi, P. & Marjomäki, T.J. (2013) Fish stocks in Lake Konnevesi during 1978–2010.

*Riista- ja kalatalous – Tutkimuksia ja selvityksiä*, 5/2013, 1-29 (in Finnish with English abstract).

Ventelä, A.-M., Tarvainen, M., Helminen, H. & Sarvala, J. (2007) Long-term management of Pyhäjärvi (southwest Finland): eutrophication, restoration – recovery? *Lake and Reservoir Management*, 23, 428–438.
