# Supplementary material for: Phenotypic plasticity in growth and fecundity induced by strong population fluctuations affects reproductive traits of female fish
Source: Ecol Evol. 2016 Jan 11;6(3):779–90. doi: 10.1002/ece3.1936 (PMC4739574; doi:10.1002/ece3.1936)
Supplement: Supplementary file 2 — Appendix S2. Total number of fish, eggs and hatched larvae in experiments and measurements in different incubation periods in 2004–2007. [file ECE3-6-779-s002.docx]

Supporting information Appendix S2. Total number of fish, eggs and hatched larvae in experiments and measurements in different incubation periods in 2004-2007. Egg _INC_ = number of eggs in incubation experiment; Egg _FFAIL_ = number of eggs in fertilization failure determination; Egg _WM_ = number of eggs in wet mass determination; Larvae _HATCHTL_ = number of hatched larvae in total length determination; Larvae _HATCHWM_ = number of hatched larvae in wet mass determination; Larvae _REARED_ = number of larvae in rearing experiment; Larvae _TEST_ = number of larvae in swimming challenge test; Larvae _TL_ = number of larvae in total length determination; Larvae _WM_ = number of larvae in wet mass determination.

|  | Lake I Pyhäjärvi | | | Lake II Pyhäselkä | | | Lake III Puulavesi | | | Lake IV Konnevesi | |  |
| --- | --- | --- | --- | --- | --- | --- | --- | --- | --- | --- | --- | --- |
|  | 04-05 | 05-06 | 06-07 | 04-05 | 05-06 | 06-07 | 04-05 | 05-06 | 06-07 | 04-05 | 05-06 | 06-07 |
| Incubation experiments |  |  |  |  |  |  |  |  |  |  |  |  |
| Females | 9 | 9 | 8 | 9 | 9 | 9 | 9 | 9 | 9 | 8 | 9 | 9 |
| Males | 27 | 27 | 24 | 27 | 27 | 27 | 27 | 27 | 27 | 24 | 27 | 27 |
| Egg _INC_ | 8 432 | 4 562 | 3 184 | 7 433 | 9 726 | 3 326 | 12 815 | 7 858 | 7 330 | 577 | 1 557 | 6 091 |
| Egg _FFAIL_ | 3 149 | 1 845 | 2 000 | 2 628 | 1 586 | 1 112 | 2 561 | 1 605 | 1 431 | 1 422 | 1 528 | 998 |
|  |  |  |  |  |  |  |  |  |  |  |  |  |
| Egg _FM_ | 50 | 50 | 50 | 50 | 50 | 50 | 50 | 50 | 50 | 50 | 50 | 50 |
| Larvae _HATCHTL_ | 85 | 57 | 32 | 85 | 90 | 51 | 89 | 90 | 90 | 19 | 20 | 35 |
| Larvae H_ATCHWM_ | 85 | 57 | 32 | 85 | 90 | 51 | 89 | 90 | 90 | 19 | 20 | 35 |
|  |  |  |  |  |  |  |  |  |  |  |  |  |
| Fecundity |  |  |  |  |  |  |  |  |  |  |  |  |
| Females | 42 | 38 | 33 | 57 | 42 | 52 | 23 | 76 | 60 | 17 | 4 | 31 |
|  |  |  |  |  |  |  |  |  |  |  |  |  |
| Hatched larvae |  |  |  |  |  |  |  |  |  |  |  |  |
| Larvae _REARED_ | 325 | 260 | 130 | 325 | 260 | 130 | 195 | 130 | 195 | - | 130 | 195 |
| Larvae _TEST_ | 125 | 62 | 40 | 125 | 59 | 40 | 75 | 36 | 55 | - | 36 | 55 |
| Larvae _TL_ | 125 | 62 | 40 | 125 | 59 | 40 | 75 | 36 | 55 | - | 36 | 55 |
| Larvae _WM_ | 230 | 180 | 40 | 157 | 156 | 40 | 112 | 108 | 55 | - | 89 | 55 |
|  |  |  |  |  |  |  |  |  |  |  |  |  |
